# Sero-prevalence of Newcastle disease and associated risk factors in chickens at backyard chicken production Kindo Koisha, Wolaita zone, Southern Ethiopia

**DOI:** 10.3389/fvets.2022.1089931

**Published:** 2023-01-09

**Authors:** Wondimu Wodajo, Nejib Mohammed, Ephrem Tora, Wasihun Seyoum

**Affiliations:** ^1^Wolaita Sodo Regional Veterinary Laboratory, Department of Microbiology, Wolaita Sodo, Ethiopia; ^2^Department of Animal Sciences, Arba Minch University, Arba Minch, Ethiopia

**Keywords:** backyard chickens, district, Newcastle disease, seroprevalence, Wolaita

## Abstract

Newcastle disease (ND) is a serious infectious disease of poultry caused by virulent strains of Avian Paramyxovirus-1 and has a substantial impact on villages where people's livelihood depends upon poultry farming in several developing countries including Ethiopia. In the district of the study area, no previous studies have been conducted. Thus, the aim of the present study was to estimate individual and household flock level seroprevalence and risk factors for ND in unvaccinated backyard chickens in Kindo Koisha district, Wolaita zone, Ethiopia. A cross-sectional study design was conducted. For the study, household flocks were sampled as a cluster, and backyard chickens within the cluster with an age of more than 3 months were sampled. A total of 598 blood samples were collected from 86 household flocks during the study period. Serum samples were tested for ND antibodies using an Indirect-Enzyme Linked Immuno Sorbent Assay. The overall seroprevalence of ND virus at individual and flock level was 17.06% (95% CI: 14.25–20.29%) and 73.26% (95% CI: 62.79–81.64%), respectively. The ND seropositivity and associated risk factors were assessed at the individual bird and flock level by using ordinary and mixed effect logistic regression, respectively. Ordinary logistic regression revealed that crossbreed chickens had significantly higher odds of ND seropositivity than local breeds, with an odds ratio of 2.15 (95% CI: 1.54–3.00; *p* < 0.001). The odds of ND seropositivity was significantly higher in backyard chickens which belongs flock size >9 in comparison to <9 with an odds ratio of 3.7 (95% CI: 1.12–12.30; *p* < 0.031). The potential flock level risk factors related to ND seropositivity in this study were flock size, chicken house cleaning frequency, water source for chickens, dead chicken disposal practice, and distance to the next neighbor household, mixing with wild birds and owning pets. In conclusion, the current study generates significant information on the seroprevalence and the potential risk factors associated with ND at individual and flock level in Kindo Koisha district, Wolaita zone. Consequently, ND vaccination campaigns should be launched, and effective extension programs should also be provided to raise awareness about the disease.

## 1. Introduction

In developing countries, rural poultry production plays a significant contribution to the economy particularly supporting the rural community (1). Taking advantage of the large proportion of poultry in these countries is a good way to supplement farmers' incomes because it requires minimal space and investment, and can be conducted by less skilled farmers (2). In Ethiopia, over 99% of the total estimated chickens are produced by village poultry, while only 1% come from intensive exotic breeds reared under intensive management systems (3). For a long time, chickens have been reared in villages for common purposes, such as egg production, meat production, and sale (4, 5). Conversely, production performance is affected by various constraints (6, 7).

The constraints that limit prospective village chicken production in Ethiopia include poor management, low inputs for feed supply, the existence of various diseases, lack of appropriate biosecurity and breeding programs (4). Regarding poultry diseases, Newcastle disease (ND) is an infectious disease that hampers poultry production, individually or concurrently in a village, in the country. Newcastle disease is a highly contagious viral disease that can infect both wild and domestic birds. ND is considered to be the most significant cause responsible for reducing both the number and productivity of chickens (8). Intensification is a contributing factor leading to the rapid spread of virulent strains between and within poultry farms. Assuming that the spread of these strains is a major threat to backyard chickens (1).

A virulent strain of avian paramyxovirus type 1 (APMV-1), known as Newcastle disease virus (NDV), belongs to the family Paramyxoviridae, is responsible for Newcastle disease (ND) (9). Based on Diel et al. (10) classification, Ethiopian Newcastle disease virus phylogenetic analysis of the 260 fragments of the fusion gene of all 29 sequenced isolates revealed that the viruses belong to the new subgenotype VIf class II virus group, and grouped with NDVs previously identified from village chickens in Ethiopia (11–14). This subgenotype was also confirmed by analysis of complete coding region of the F gene of the representative viruses and showed 95–99.3% similarity (11, 15). Chaka et al. (14) reported that most viral strains found in village chickens in Ethiopia are virulent. In essence, bird species appeared to be susceptible to infection by both high and low virulence for chickens with APMV-1, although clinical symptoms vary greatly and depend on a variety of factors, including the virus, the host species, the age of the host, infection with other organisms, environmental stress, and the immune status of the infected bird (9).

Globally, the main transmission sources of Newcastle disease include migratory birds and the trade in live birds, which are also significant risk factors for transmission within endemic areas. Backyard poultry is the main poultry production sector that suffers greatly from ND. This is related to the low level of biosecurity measures and the inadequate supply of vaccinations. For instance, Newcastle disease was alleged to be the causative agent of roughly 90% of backyard poultry mortality in Ethiopia, from 1993 to 2021 (16–18). Even though backyard poultry are important in Ethiopia, relatively little is known about NDV prevalence in general and the current study area in particular. There is a shortage of information, with almost no published reports of ND in the current study area. Furthermore, due to a lack of routine surveillance programs in the study District, there is a need to consider the sero-prevalence of NDV in apparently healthy birds and the risks associated with ND occurrence. To fill this gap, a cross-sectional survey was carried out in 2021 to estimate the prevalence and associated risk factors in backyard poultry in Kindo Koisha.

## 2. Materials and methods

### 2.1. Study area description

Kindo Koisha, a western district in Wolaita zone, is 410 kilometers south of Addis Ababa, between the coordinates 6° 49' 59.99” N Latitude and 37° 29' 59.99” E Longitude. The altitude range in the Koisha district spans from 700 to 1,750 mean above sea level and has an average annual rainfall of 900 mm. The district comprises ~15% of Wolaita's total layer population. Currently, there are 214,167 poultry (149,917 local and 64,250 cross/exotic breeds), which are reared under a backyard management system. Kindo Koisha has a renowned natural wetland, which is visited by migratory birds. The presence of backyard poultry farms and commercial poultry farms along migratory birds' fly-paths may promote an opportunity for exchange of NDV among bird populations.

### 2.2. Study population

The study animals were all apparently healthy chickens with a history of no vaccination in the Kindo Koisha district of Wolaita zone, southern Ethiopia. A sample of backyard chickens was taken from the district, taking into account their breed (local and cross), age, and sex. Apparently healthy chickens with no vaccination history and chickens with an age above 3 months (> 3 months old) were included. Participants included in this study were all chicken producers, both local and crossbred, in extensive/backyard production systems. Chickens appearing to be healthy with a history of vaccinations and those with ages below 3 months managed under an intensive production system were excluded. Also, exotic breeds were excluded from the current study.

### 2.3. Study design

A cross-sectional study was conducted from January 2021 to May 2021 to estimate the individual chicken and household flock level seroprevalence of Newcastle disease and associated risk factors in backyard poultry in the selected districts of Wolaita zone, southern Ethiopia. Moreover, a checklist was employed to assess the likely risk factors contributing to Newcastle disease in backyard chickens and the perceptions of poultry owners about the disease.

### 2.4. Sampling techniques and sample size determination

To begin, the study area was purposely selected for its substantial backyard poultry populations and the amount of household flocks kept under the backyard production system. The administrative district, Kindo Koisha has 26 Kebeles. The district's Livestock and Fisheries Resource Development Offices provided a list of kebeles in the district. Households with backyard poultry flocks were listed in each kebele, and 86 household flocks were selected randomly. All backyard chickens over the age of 3 months were sampled from each household flock that was reared in a free-range system. They were free to roam and feed themselves throughout the day and returned to their enclosures for shelter and supplements on occasion.

The sample size required was calculated based on expected 50% NDV seroprevalence with 95% confidence and 5% precision. Because each household was selected as a cluster in this study, the sample size was determined using a one-stage cluster sampling method (19), in which all backyard chickens aged more than 3 months old in the household flocks were randomly selected with a design effect of 3 for clustering effect adjustment based on a previous study by Chaka et al. (13). In a report on the characterization of village chicken production and marketing systems in Gomma district, Jimma zone, Molla et al. (20) reported a mean flock size per household of 6.2 birds. As a result, the average flock size per household in the current study was estimated to be 6.2 birds.

The appropriate formula of one-stage cluster sampling for a 95% confidence interval is then:


$$\begin{array}{l} g=\frac{-1.962\;\times D\;\times \;P\;\exp\;\left(1-P\exp\right)}{nd^{2}} \end{array}$$

where:

g = number of clusters to be sampled; *n* = predicted average number of animals per cluster (*n* = 6.2); Pexp = expected prevalence (Pexp = 50%); d = desired absolute precision (d = 5%); D = Design effect (an adjustment factor used to allow for clustering).

Therefore, the number of household flocks (g = 86) and the number of chickens (86 × 6.2 = 533 backyard chickens) were sampled for seroprevalence. The intended number of household flocks and backyard chickens to be sampled was then proportionally stratified by districts and kebeles, as stated in Table 1. This was based on the estimated number of household flocks and domestic poultry present in the district and each kebele.

**Table 1:** Population of chickens and number of households per kebele.

**District**	**Kebele**	**Households with flocks**	**Chickens**	**Mean flock size**	**Household flocks sampled**	**Chickens sampled**
Kindo Koisha	Dada Kare	409	3,795	9.28	19	119
	Fagena Mata	283	3,462	11.46	13	109
	Molticho	217	2,322	10.7	20	73
	Mundena	403	5,104	12.33	18	154
	Manara	432	4,571	10.68	16	143
Total		1,744	21,254		86	598

### 2.5. Data collection and procedures

A semi-structured questionnaire was administered to households in the study sites to collect survey data through in-person interviews. This survey was used to support a cross-sectional investigation and was conducted after carefully explaining the purpose of the study to the interviewees. From each kebele, 86 backyard chicken owners were selected from households in that district. The sample size of the candidates was determined according to the formula (*n* = 0.25/SE2). Therefore, 86 households were selected using the standard error (SE) of 0.05(4%) with 95% confidence interval.

### 2.6. Laboratory procedures

#### 2.6.1. Sera collection and testing

A 1.5-3 ml blood sample was obtained from the humeral region brachial wing vein using a 3 ml syringe and needle. After that, the blood-filled syringe was held horizontally until it clotted. After clotting, serum was collected in a syringe that had already been tagged with a marker pen. The serum was separated and decanted into labeled cryovial tubes, and stored at −20°C until an enzyme-linked immunosorbent test was performed at Sodo Regional Veterinary Laboratory, Ethiopia.

#### 2.6.2. Indirect enzyme-linked immunosorbent assay (ELISA)

An indirect ELISA technique was carried out through the use of ID.vet Innovative Diagnostics kits (IDvet, 310, rue Louis Pasteur-Grabels-France: ID Screen^®^Newcastle Nucleoprotein Indirect Version 2) and was designed to detect antibodies directed against Newcastle Nucleoprotein (NDVNP). Assays were conducted following the manufacturer's recommended procedure. Multiskan EX (Lab Systems) ELISA microplate reader was used to read and record optical density (OD) values at an absorbance filter of 450 nm (21).

Using a microplate reader, the optical densities (ODs) at 450 nm were read and quantified. The color intensity was directly related to the amount of antibody present in the sample. Based on the ODs the sample to positive (S/P) ratios were calculated and used to express the mean (S/P) ratio per group. Samples with antibody levels above the thresholds defined by the kit manufacturer were classified as positive; all other samples were classified as negative. Validation checking was also conducted: the test was deemed to be valid if the mean OD value of positive control (OD_PC_) is >0.25 and the ratio of the mean values of the positive control OD (OD_PC_) to the negative control OD (OD_NC_) is >3. For each sample, the sample to positive ratio (S/P) and antibody titer was calculated. The results were interpreted as: *S*/*P*-value ≤3 and ELISA antibody titer ≤993 for negative ND immune status; *S*/*P*-value >3 and ELISA antibody titer >993 for positive ND immune status (21). The samples were classified into positive and negative based on the comparison of the absorbance between the samples and the thresholds defined by the kit's manufacturer.

The antigen and conjugate dilution was fixed at 1:200 and 1:1,600, respectively, as these dilutions gave the highest ratios of absorbance with positive anti-NDV serum divided by absorbance with negative serum (22) and Kit's manufacturer.

### 2.7. Data management and analysis

Data generated through the serological study and questionnaire surveys were analyzed by using STATA Version 16.0. Initially, descriptive statistics was employed to summarize the percentage of both dependent and independent variables. One-way analysis of variance (ANOVA) was also used to compare the means of titres in kebeles. Seroprevalence estimate for antibodies against NDV was computed as percent of seropositive chickens from the total number of animals examined. Flock-level prevalence was calculated when at least one bird within a household tested positive for ND antibodies.

A univariable ordinary and mixed effect logistic regression was run to observe the unconditional association between ND seroprevalence and flock composition at the individual and household levels, respectively. Considering a liberal *p*-value (*p* = 0.25), potential predictors were selected for multicollinearity assessment using Goodman and Kruskal's Gamma values. All the non-collinear predictors (gamma values between −0.6 and +0.6) were subjected to multivariable logistic regression. The final model was developed using the backward elimination technique, based on the likelihood ratio test and Wald's statistics (*p* < 0.05). Also, any potential interactions between predictors were tested by forcing statistically significant variables into the multivariable model and examining changes in ORs and *p*-values of the main effects. By measuring odds ratios, associations between exposure variables and ND seroprevalence were further determined. Confounding effects of predictors were also checked by using the changes in the proportion of OR. Then, a covariate variable was considered as a confounder and included in the model if its inclusion changed the OR of the estimated risk at least by 25% (23). As a final step, Hosmer and Lemeshow statistics were used to assess the goodness-of-fit and the receiver operating curve for reliability of the model (24).

## 3. Results

### 3.1. ND seroprevalence at individual and household flock level

During the study period, data on individual chickens and household flocks were collected from five randomly selected kebeles of the Kindo Koisha district, Wolaita zone (Table 2). In contrast to the individual chicken level seroprevalence of NDV of 17.05% (95% CI: 14.24–20.29%), the flock level of ND was 73.26% (95% CI: 62.79–81.64%). On kebele level, the highest ND seroprevalence was observed in Molticho kebele (35.62%) and the lowest in Dada Kare kebele (10.92%) (Table 2).

**Table 2:** Newcastle disease seroprevalence at individual and household flock level.

**Districts**	**Kebele**	**Seroprevalence (%)**	
		**Individual diseased (%)**	**Flock level diseased (%)**
Kindo Koisha	Dada Kare	13/119 (10.92%)	9/19 (47.42%)
	Fagena Mata	23/109(21.10%)	9/13 (69.23%)
	Molticho	26/73 (35.62%)	7/20 (35.21%)
	Mundena	20/154(12.99%)	10/18(55.56%)
	Manara	20/143 (13.99%)	8/16 (50.12%)
Agro-ecology	Mid land	10/64 (15.63%)	–
	Low land	92/534(17.23%)	–
Grain supplement	Provided	48/342 (14.04%)	48/70 (81.40%)
	Not provided	54/256 (21.09%)	15/16 (18.60%)
Dog owning	Yes	60/279 (20.20%)	8/21 (24.42%)
	No	42/301 (13.94%)	55/65 (75.58%)
Mixing with wild bird	Mixed	83/440 (18.86%)	60/77 (89.53%)
	No mix	19/158 (12.03%)	3/9 (10.47%)
Contact with neighbor flocks	Yes	76/327 (23.24%)	58/76 (88.37%)
	No	26/271 (9.59%)	5/10 (11.63%)
Dead poultry disposal practice	Thrown nearby	74/332 (22.29%)	42/51 (65.63%)
	Thrown afar	14/209 (6.70%)	21/32 (33.33%)
	Buried or Burnt	14/57 (24.57%)	0/3 (0.00%)
Water source	Closed: tap water	28/272 (10.29%)	16/32 (25.40%)
	Open: pond/ river	74/326 (22.07%)	47/54 (74.60%)
Poultry waste disposal practices	Thrown nearby	74/334 (22.16%)	54/72 (85.71%)
	Thrown far away	28/264 (10.61%)	9/14 (14.29%)
Cleaning frequency of poultry farm	Daily basis	32/311 (10.29%)	13/21 (20.63%)
	<1 per week	14/71 (19.72%)	44/59 (69.84%)
	Weekly basis	56/216 (26.93%)	6/6 (5.62%)
Flock restocking	From other sources	22/120 (18.33%)	53/65 (84.13%)
	Own its own	80/487 (16.74%)	10/21 (15.87%)
Flock replacement	Hatching at home	10/142 (7.04%)	1/6 (1.59%)
	From local market	62/283 (21.9%)	50/56 (79.39%)
	Conjointly	30/173 (17.34%)	12/24 (19.07%)
Stage of chicken	Pullet	14/95 (14.74%)	–
	Cockerel	9/47 (19.15%)	–
	Hen	52/329 (15.81%)	–
	Cock	27/127 (21.26%)	–
Flock composition	Local only	40/293 (13.65%)	–
	Mixed	62/305 (20.33%)	–
Flock size	≤ 9 chickens	53/337 (15.73%)	41/60 (66.13%)
	>9 chickens	49/261 (18.77%)	21/25 (33.87%)
Chicken breed	Local	73/500 (14.60%)	–
	Cross	29/98 (29.59%)	–
Sex of chicken	Male	35/170 (20.59%)	–
	Female	67/428 (15.65%)	–
Age group	Chicks: 3–6 months	23/135 (17.04%)	–
	Adult: 7–16 months	63/365 (17.26%)	–
	Old: ≥16 months	16/98 (16.33%)	–
Total		102/598 (17.06%) (CI: 14.25–20.29)	63/86 (73.26%) (95% CI: 62.79–81.64%)

The number of chickens sampled in Kindo Koyisha district from backyard scavenging poultry was 598. One Hundred Two of these chicken serum antibodies were considered to be positive for NDV. The proportion of seropositive chickens for NDV was 23/135 (17.04%), 63/365 (17.26%), and 16/98 (16.33%), respectively, for young, adult and old chickens. Moreover, chicken breed was classified as local and crossbred, and a higher number of crossbred chickens were seropositive 29/98 (29.59%) from samples than local breeds 73/500 (14.60%). For the flock restocking, NDV seropositive chickens were 22/120 (18.33%) from other sources and 80/487 (16.74%) from their own source. Furthermore, chicken flock replacement was classified as hatching at home, bought from local market and conjoint source were seropositive 10/142 (7.04%), 62/283 (21.9%) and 30/173 (17.34%), respectively.

### 3.2. Comparison of antibody response between kebele and chicken in breeds

**Figure 1:**
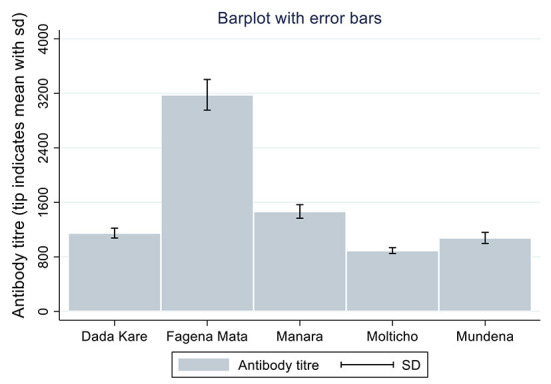
Comparison of antibody response in different peasant association (kebeles).

**Figure 2:**
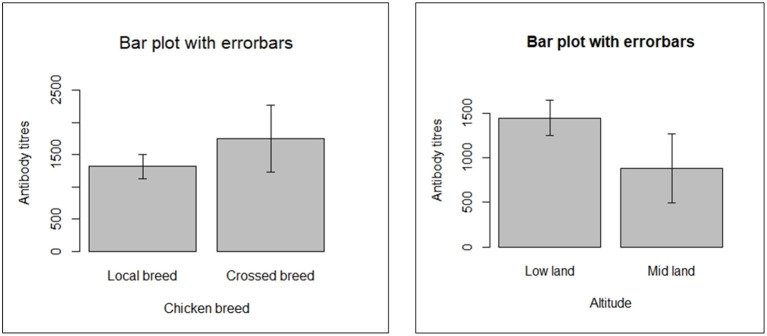
Comparison of antibody response in different chicken in breeds.

The antibody titres recorded in the present study ranged from 3.46 (negative for NDV antibody) to 11,823 (positive for NDV antibody). The mean antibody titer for backyard chickens sampled in the study was documented to be 1,386.04. Means of NDV antibody titres for the five kebeles of the district are shown (Figure 1). There were differences in mean antibody titres between breeds, kebeles and agro-ecological zone among backyard chickens, as illustrated in Figures 1, 2.

### 3.3. Association of risk factors with ND seroprevalence

The statistical associations of chicken risk factors with ND seroprevalence were depicted (Table 3). This study considered 19 variables (6 for individual chicken level and 13 for household flock level) using logistic regression analysis. During backward selection, variables that were insignificant in the multivariable regression were removed; as a result, the main effects were included in the multivariable regression.

**Table 3:** Univariable analysis of individual level risk factors with ND seroprevalence.

**Risk factors**	**Categories**	**OR**	**95% CI**	**P-value**
District	Dada Kare	Ref		
	Fagena Mata	2.36	0.87–6.83	0.092
	Manara	1.39	0.53–3.64	0.508
	Mundena	1.44	0.54–3.83	0.459
Age (months)	Young (3–6)	Ref		
	Adult (7–16)	1.07	0.74–1.54	0.702
	Old (>16)	1.06	0.68–1.67	0.782
Sex	Male	Ref		
	Female	0.82	0.60–1.13	0.234
Breed	Local	Ref		
	Exotic	2.4	1.7–3.3	<0.001
Agro-ecology	Lowland	Ref		
	Midland	2.25	1.67–3.02	<0.001
Type/category of Chickens	Pullets	Ref		
	Cockerels	1.09	0.57–2.08	0.784
	Hens	1.04	0.69–1.57	0.858
	Cocks	1.31	0.81–2.12	0.271

#### 3.3.1. Association of individual level risk factors with ND seroprevalence

Variables significant at a liberal *p*-value (*P* < 0.25) in univariable analysis were entered into multivariable logistic regression. The final multivariable logistic regression model revealed two potentially significant explanatory variables (*P* < 0.05) associated with individual chicken level seroprevalence (Table 4). Hence, the final multivariate logistic regression for individual chicken level risk factors such as agro-ecology and breeds of backyard chickens is associated with seroprevalence (*P* < 0.05).

**Table 4:** Multivariable logistic regression for individual level risk factors.

**Risk factors**	**OR**	**95% CI**	**P-value**
**Agro-ecology**		
Low land	Ref		
Mid land	2.12	1.76–3.19	0.022
**Breed**		
Local	Ref		
Cross	2.15	1.5–3.0	<0.001

The odds of ND sero-positivity was significantly higher in backyard unvaccinated chickens belonging to Kindo Koisha's midland agro-ecology in comparison with chickens belonging to low land with an odds ratio of 2.12 (95% CI: 1.76–3.19; *p* < 0.001).

#### 3.3.2. Association of flock level risk factors with ND seroprevalence

The present study revealed ND sero-prevalence which was influenced by a variety of risk factors at household flock level ranging from the study district's peasant association to owning pets in the household.

Variables significant at liberal *P*-value 0.25 in the univariable mixed effect analysis were selected, and then tested for main effect using multivariable mixed regression. The final multivariable mixed effects logistic regression model indicated seven potentially significant explanatory variables (*P* < 0.05) associated with household flock level seroprevalence (Table 5). There were significant flock level risk factors (*P* < 0.05) with regards to the seroprevalence of ND, which were flock size, chicken house cleaning frequency, poultry water source, dead poultry disposal practice, distance to the next neighboring flock, mixing with wildlife, and owning pets.

**Table 5:** Multivariable logistic regression for Household level risk factors.

**Risk factors**	**Category**	**OR**	**95% CI**	**P-value**
Flock size	<9	Ref		
	≥9	3.72	1.12–12.30	0.031
Chicken house cleaning frequency	Daily	Ref		
	Once/twice weekly	4.27	1.16–15.74	0.029
	Less than once weekly	10.7	0.70–162.5	0.088
Water source	Closed (tap or borehole)	Ref		
	Open (pond or river)	5.8	1.93–17.6	0.002
Dead chicken disposal practice	Throwing nearby	Ref		
	Throwing far away	0.07	0.02–0.25	<0.001
	Bury/burn	0.001	0.01–0.03	<0.001
Distance to the next neighbor house flock (m)	<116	Ref		
	≥116	0.08	0.02–0.28	<0.001
Mixing with wild birds	No	Ref		
	Yes	63.77	7.20–564.76	<0.001
Owning pets (dogs and cats)	No	Ref		
	Yes	9.89	2.64–35.21	0.001

Ref, reference category; OR, odds ratio; *P* ≤ 0.05 is significantly different.

## 4. Discussion

This study disclosed the prevalence of circulating NDV in backyard poultry in Kindo Koisha district, Wolaita Zone, Southern Ethiopia. In Ethiopia, 89% of the total chicken population is managed under backyard poultry production systems (25). In backyard chicken production, farmers usually own indigenous chicken breeds. The production system is comprised of scavenging feed and small-scale poultry with low productivity, and long rearing periods. Natural brooding is practiced by farmers to replace chicks, which is prone to a number of diseases that cause high chicken mortality rates due to lack of vaccination.

The overall seroprevalence of NDV at chicken level was 17.05% (95% CI: 14.24–20.29%). This finding is higher than the estimates reported in a study from the Sebata Hawas district, Oromia region (11.34%) (4); Kersana-kondalaity district, Ethiopia (5.6%) (26); Eastern Shewa zone, Ethiopia (5.9%) (5) in a seroprevalence study of Newcastle disease in backyard chickens. This result was higher than that studies abroad, 11.7% in the Indian state of Odisha (27) and 9.8% in Haryana, India (28). Compared with the prevalence estimate in this study, the prevalence in poultry farms in Oman (33.8%) (29); in the Moyamba district of Sierra Leone (56.4%) (30) was higher. The seroprevalence estimate at chicken level in the present study was almost identical to the seroprevalence estimate for backyard chickens in Bishoftu, Ethiopia (31), which reported a seroprevalence of (23.4%); in Ivory-Coast (22%) (32). The difference might be due to variation in geographical locations, degree of previous exposure to the pathogen, agro-ecology of different study areas, environmental factors and method of virus identification could influence the epidemiological triad of NDV transmission.

In this study, the household flock seroprevalence of NDV was reported to be 73.26% (95% CI: 62.79–81.64%), which was significantly higher than previously reported cross-sectional studies on backyard chickens in Ethiopia's eastern Shewa zone (17.4% in wet season and 27.4% in dry season) (5). This indicates that study district have significant numbers of chickens that would be highly susceptible to NDV infection, as NDV is extremely transmissible among such flocks (9). From Chittagong, Bangladesh, Belgrad et al. (33) reported a lower prevalence of ND at the household level (31.8%). From Oman, Alsahami et al. (29) reported a lower (57.1%) sero-prevalence of NDV at flock level than the present report. However, authors reported higher flock-level seroprevalence in Southern Brazil (34) (87.5%), Oman (8) (90%) and Algeria (35) (82.69%). The difference in results might be associated with geographical locations, diagnostic techniques, climatic conditions, poor biosecurity, lack of vaccination, contact with neighboring household birds and unhygienic feeding practices.

Fagena Mata kebele had the highest mean antibody titer of 3,178 while Molticho kebele had the least mean titer of 893. The results of this study agree with those of a study conducted by Osman et al. (36) on backyard chickens in Somalia where variation was found across administrative borders, as they found higher levels of mean NDV antibody titer at district level between six districts. Moreover, authors revealed mean of NDV antibody titres for the five districts (Wadajir district had the highest mean antibody titer of 8,517 while Hodan district had the least mean titer of 471 and mean titres for Dharkenley, Hawlwadag, and Daynile districts ranged between 3,000 and 5,000). It could be assumed that the chickens could withstand ND outbreaks and subsequently acquired high levels of immunity that prevented them from developing clinical signs of the disease (37). The high ND antibody titres recorded in the current study indicated that there is circulation of field strains of ND virus in the study area.

The presence of NDV in serum and tracheal samples indicates that chickens have previously been exposed to the virus (37, 38). Chicken sera in this study contained antibodies to Newcastle disease virus, indicating past exposure to the virus. Since all of the chickens sampled were over 3 months of age, this also reveals chickens' exposure to natural infection. This is according to Alexander (39) and Rezaeianzadeh et al. (40), who reported that the presence of anti-NDV antibodies on farms with unvaccinated animals might be related to exposure previously to natural NDV infection. Typically, it is confirmed that maternally derived NDV antibodies provide protection to chickens against ND up to two weeks of age. The rate of declination of maternally derived antibodies was about half every 7 days (41–45).

This study finding was influenced by a variety of putative risk factors at chicken level for breeds of chicken and agro-ecology in univariable analysis and chicken breed for multivariable analysis. It was indicated that the seroprevalence of NDV in the midland was 2.12 times higher compared with the lowland (Table 6). This contradicts the findings by (7) and (2) where low altitudes have high seroprevalence, though there are few chickens living in the highland and they thought that the number of chickens is a factor in the transmission of the disease. Njagi et al. (37) studied the prevalence of Newcastle disease virus in village indigenous chickens in varied agro-ecological zones in Kenya and they revealed higher seroprevalence (17.8%) in the dry hot zone (lower midland) compared to the cool wet zone (lower highland) seroprevalence of 9.9% although the difference was not statistically significant (*P* > 0.05). The discrepancy in the rates of NDV antibodies in the midland and lowland might be because of ecological variations in NDV activity. This may perhaps be a reflection of the impact of the environment on the viability of NDV and its epidemiology. These variations could be explained by differences in study settings or by exposure to mild virus strains that induced immunity but did not kill many chickens (5).

**Table 6:** Univariable mixed effects logistic regression of flock level risk factors with ND seroprevalence.

**Risk factors**	**Level/category**	**OR**	**95% CI**	**P-value**
Peasant association	Dada Kare	Ref		
	Fagena Mata	2.36	0.87–6.83	0.092
	Manara	1.39	0.53–3.64	0.508
	Mundena	1.44	0.54–3.83	0.459
Flock size	<9	Ref		
	≥9	1.4	0.7–2.7	0.313
Flock replacement	Conjointly	Ref		
	Hatching at home	0.06	0.007–0.48	0.008
	Buying from local market	15.63	6.04–40.40	>0.001
Poultry house cleaning frequency	Daily	Ref		
	Once/twice weekly	2.03	1.0–4.2	0.059
	Less than once weekly	11	1.3–93.4	0.028
Poultry waste disposal practice	Thrown nearby	Ref		
	Thrown far away	0.32	0.13–0.79	0.014
Water source	Closed (tap or borehole water)	Ref		
	Open (pond or river)	6.07	3–12.3	<0.001
Dead poultry disposal practice	Thrown nearby	Ref		
	Thrown far away	0.19	0.09–0.4	<0.001
	Buried/burnt	0.02	0.003–0.2	0.001
Contact with neighboring flocks	No	Ref		
	Yes	5.30	1.9–14.71	0.001
Distance to neighboring flock (meters)	<116	Ref		
	≥116	0.11	0.05–0.23	<0.001
Mixing with wild birds	No	Ref		
	Yes	4.81	1.72–13.47	0.003
Own pets	No	Ref		
	Yes	7.02	3.12–15.79	<0.001
Grain supplement provided	No	Ref		
	Yes	0.27	0.12–0.60	0.001

It was shown in the multivariable mixed logistic regression that the odds of ND seropositivity of crossbreed backyard chickens was significantly higher than chickens categorized as local breed with an odds ratio of 2.15 (95% CI: 1.54–3.00; *P* < 0.001). Therefore, the ND seropositivity of crossbreed chickens was almost 2 times higher than local breed backyard chickens. In line with this study, Endalew (46) reported a seroprevalence of 2.13 times higher in cross breeds than in local ones. This indicates a significant difference between local and cross-breed chickens. A study conducted by Tulu (4) indicated variation in odds of the seroprevalence of Newcastle disease among cross and local breeds of chickens but the difference was not statistically significant. This discrepancy in breed differences may be related to differences in management practices in backyard production systems between this study and previous studies. Vui et al. (47) reported an insignificant difference (*P* > 0.05) in the seroprevalence between indigenous (local) and cross breeds of chickens. However, variation could be caused by multiple factors, including management, season, and unequal proportions of breeds sampled.

The odds of ND seropositivity was significantly higher in poultry house cleaning frequency of once or twice weekly compared with daily cleaning frequency (OR = 4.27; 95% CI: 1.16–15.74; *P* = 0.029). As it was indicated in the multivariable mixed model, daily cleaning was a protective factor compared with once or twice weekly cleaning. Thus, the present study indicated that daily poultry house cleaning reduced the chance of transmission and decreased ND seropositivity. This study coincides with Chaka et al. (13) who reported poultry house cleaning frequency as a risk factor. It has been established that NDV can survive in poultry feces for several days to weeks depending on temperature (48, 49). Despite that, a study reported from Chittagong, Bangladesh, indicated that Newcastle disease seroprevalence in rural poultry indicated that daily cleaning increased the transmission of the virus due to disturbing the litter of infected chickens. Thus frequent disturbance of feces with NDV may supposedly increase the NDV infection. However, the study did not consider NDV harbored within the feces or stress to the birds caused by cleaning. According to Belgrad et al. (33) frequent cleaning may produce excessive stress on poultry predisposing them to infection.

When people used open water sources, such as ponds or rivers, to drink water for their chickens, the odds of seropositivity were higher than when they used closed water sources (tap or borehole water) (OR = 5.8; *P* = 0.002). The increased ND seropositivity in household flocks that used water from open water sources (pond or river) could be attributed to contamination of water by wild birds that then transmitted the NDV to household flocks in the study area. It was ostensibly disclosed by Chaka et al. (13) that using open water sources increased the risk of incidence of ND in household flocks, more specifically in households that used ponds and rivers. These water sources, unlike tap or borehole water, are easily contaminated by droppings from wild birds or other poultry, which may be carriers of NDV and undoubtedly, our study supports this point.

The odds of ND seropositivity in the household flocks practicing the dead chicken disposal by throwing carcasses of dead chickens far away may decrease by 93% less likely in comparison with the households throwing nearby (OR = 0.07; 95% CI: 0.02–0.25; *P* < 0.001). Essentially, the households burying/burning the dead chicken carcasses as dead bird disposal practice was very less likely to be ND seropositive (0.0013; 0.0001–0.0340; *P* < 0.001) when compared with dead bird disposal practice of throwing nearby. Despite being statistically insignificant, Chaka et al. (13) considered the practice of disposing of dead birds as a risk factor. In this study, however, it was a significant risk factor associated with NDV seropositivity. Similarly, Belgrad et al. (33) found that dead bird disposal practices with ND seropositivity were significantly associated (*p* = 0.04) but categorizing the practices showed slight variations: bury and throw in pit, feed to other animals, throw in pond/lake/canal or sea, and throw in bushes/road side.

Based on the distance from the next neighbor's backyard chicken flock (>116 m), there is a 92% lower probability of ND seropositivity in the households categorized within the distance (>116 m) compared to the households within (116 m) (OR = 0.08; 95% CI: 0.02–0.29; *P* < 0.001). Chaka et al. (5) stated that the closer the distance, the greater the likelihood of mixing flocks from different households and the spread of disease. Considering distance to the neighboring household flock as a risk factor is in line with Alexander (39) who listed airborne spread as one mechanism for the spread of NDV. An alternative explanation for the identification of distance to nearest neighbor poultry farm as a risk factor might be a significant number of horizontal contacts between farms that are close together including the movement of people, vehicles and fomites between farms. Such movements have been identified as a potential route of transmission for NDV.

Backyard chickens that mixed with wild birds have increased odds of ND seropositivity by 63.7 times over chickens that weren't allowed to mingle with the wild birds scavenging in the environment (OR = 63.77; 95% CI: 7.20–564.76; *p* < 0.001). Since backyard poultry are more likely to be in contact with migratory wild birds due to the lack of biosecurity measures, they can serve as sentinels for pathogen introduction, like NDV, and dissemination within a region and to commercial poultry flocks (50, 51) which coincides with the report of this study. As the role of wild birds is well-known potential for the transmission of NDV, it could be hypothesized that they might serve as agents of NDV introduction and spread of the disease. A study conducted in China revealed that contact with wild birds was associated with an increased risk of infectious diseases in backyard poultry (50).

Households that owned pets along with flocks may result in odds of ND seropositivity 9.9 times increase in comparison with those that did not own (OR = 9.89; 95% CI: 2.64–35.21; *p* = 0.001). It is assumed that pets (dogs and cats) would consume and carry dead backyard chickens in the village to their homes, possibly contaminating and transmitting NDV to the scavenging backyard chickens in the household flocks. However, Chaka et al. (13) reported that the presence of pets by itself shall not be taken as a risk factor but among the households which reported to have had ND outbreaks, 22% considered it to be associated with dogs bringing in dead birds from other places and chickens scavenging on them. A targeted survey by Marks et al. (34) on ND virus in backyard poultry flocks placed in wintering sites for migratory birds from Southern Brazil showed the presence of cats and association of the increased prevalence ratio of ND in backyard poultry farmer owned pets; however, it was not statistically significant (Prevalence ratio =1.29; *p* = 0.19).

## 5. Conclusion and recommendations

The present study revealed higher seroprevalence of NCD in backayard chickens and household flocks in the Kindo Koisha district of Wolaita zone. This implies that ND is endemic and would pose a continuous threat to backyard chickens. The risk factors associated with ND occurrence at the individual chicken and household level were assessed. Peasant association and breed differences were significantly related with ND at the individual chicken level. The other potential risk factors significantly associated with the seroprevalence for ND at household flock level were flock size, chicken house cleaning frequency, water source for chickens, dead chicken disposal practice, and distance to the next neighbor house flock and owning pets. Hence, continuous sero-surveillance should be conducted in conjunction with virus isolation and genetic characterization of the circulating strains as well as risk factors-based prevention and control initiatives.

## Data availability statement

The original contributions presented in the study are included in the article/supplementary material, further inquiries can be directed to the corresponding author.

## Ethics statement

The animal study was reviewed and approved by Animal Ethics and Welfare Committee, Arba Minch University. Written informed consent was obtained from the owners for the participation of their animals in this study.

## Author contributions

WW: conceptualization, writing research, and methodology. NM: research title selection, editing, data curation, and supervision. ET and WS: revision, editing, and data curation. All authors contributed to the article and approved the submitted version.
